# Report of the Foreign Relations Committee

**Published:** 1901-03

**Authors:** 


### Foreign Correspondence.

### REPORT OF THE FOREIGN RELATIONS COMMITTEE.

To the Editor:

Dear Sir,—In the report found on page 678 of your journal for October, 1900, some passages occur relating to Germany which might be subject to misinterpretation, and I therefore beg you to give the following a place in your esteemed journal. The report says, "The clinical instruction is largely devoted to extraction and oral surgery. The practical work is usually quite limited."

It is quite true that more time is devoted to the extraction of teeth than in American colleges, if I am properly informed. It is a mistake, however, to infer from this that the other work is neglected on that account. The students at the University of Berlin take from one to two terms in extracting, and from two to three in the conservative treatment of the teeth, and a proportionate time in mechanical dentistry. The statement that the practical work is usually quite limited might have been true twenty years ago, and may be true now of a few schools, but in all the better German dental colleges at the present time the amount of time spent in practical work will be found to make a fair approach to that spent in the average American dental schools, although the need of a longer term of study is here severely felt, and it is to be hoped that ere long an extra year will be added.

The report states further that "The examinations have very little resemblance to ours, each teacher asking three questions out of a list of forty approved by government. They are not usually as exhaustive or comprehensive or scrutinizing as ours." This statement scarcely gives an adequate conception of the way in which examinations are here conducted. The examinations, which must be passed before a *State board of examiners,* are divided into four parts:

I. *Special Surgery.*—The candidate must examine a patient in the presence of the examiner, give the diagnosis, prognosis, and treatment of the case, and write a short thesis on it, which must be delivered to the examiner the next morning.

II. The second part of the examination is divided into three subjects:

1. Anatomy and Physiology.

2. General Pathology, Therapeutics, Materia Medica, and Toxicology.

3. Oral Surgery, Pathology, and Therapeutics.

In each of these three subjects the student must answer, in writing, two questions, which he draws out of a receptacle in which there are fifty questions.

III. *Practical Dentistry.*—In the third part of the examination the student has to show his practical ability in the operative and mechanical branches of dentistry.

1. In the operative department he must demonstrate the manipulation of the different dental instruments upon a patient, insert at least two fillings, one of which must be gold, treat a root-canal, extract two teeth, and clean the teeth of one patient,—all in the presence of the examiner.

2. In the mechanical department he must make and fit in the mouth of a patient at least one artificial denture or a regulating apparatus. The examiners may extend the practical examinations *ad libitum.*

IV. The fourth part of the examination is oral, held before three examiners, one of whom must be a graduated German dentist (Zahnarzt). The subjects in particular are Anatomy, Physiology, Pathology (general and special), and Materia Medica. But questions may be asked on any subject which comes within the range of dental science.

When the candidate passes these examinations successfully, he receives his diploma, not from the dental school or university, but from the Ministry of Education, which entitles him to practise as "Zahnarzt" in Germany.

We recognize the fact that dentistry has been making rapid strides in America during the last few years, and we rejoice over it, but at the same time we have not been quite inactive here; and I am not sure but that the *progress* made in Germany during the last ten years might compare not unfavorably with that made in America during the same period. What we here most lack, and what we hope to obtain in course of time, is the splendid equipment of the better American schools.

Very sincerely yours,

W. D. Miller.

[The foregoing from Professor Miller was referred to Dr. W. C. Barrett, Chairman of the Foreign Relations Committee, and the following reply was sent by him for publication.—Ed.]

That to which Professor Miller refers in his communication is the last annual report of the Foreign Relations Committee of the National Association of Dental Faculties. Some time before that report was made Professor Miller, as the Chairman of the Advisory Board for Germany, had sent an account of the methods pursued in the examinations of the University of Berlin, with a summary of the instruction given in the Dental Department. This being but one of the very many communications received from foreign countries about that time, it was unfortunately mislaid and lost, nor has it yet been found. When, therefore, just in time for use, another statement was sent in by some one in whose knowledge and candor the committee had confidence, it was substituted for the official one. It did not pretend to do more than attempt to give a summary of the practical results and workings of the examinations, but to the impartiality of the writer the committee believes it can confidently bear witness. The members deeply regret if any injustice has been done, as they have tried very hard to be at all times and in all things fair and free from prejudice. The blame, if any there is, rests wholly with the Chairman, who is custodian of the records.

W. C. Barrett,

*Chairman Foreign Relations Committee.*

